# Ultrasonography for non-invasive sex identification and reproductive assessment in Nile tilapia (*Oreochromis niloticus*)

**DOI:** 10.3389/fvets.2024.1467158

**Published:** 2024-10-09

**Authors:** Piyathip Setthawong, Matepiya Khemthong, Tuchakorn Lertwanakarn, Win Surachetpong

**Affiliations:** ^1^Department of Physiology, Faculty of Veterinary Medicine, Kasetsart University, Bangkok, Thailand; ^2^Department of Veterinary Microbiology and Immunology, Faculty of Veterinary Medicine, Kasetsart University, Bangkok, Thailand; ^3^Laboratory of Biotechnology, Chulabhorn Research Institute, Bangkok, Thailand

**Keywords:** sex identification, tilapia, ovary, testis, ultrasound, aquaculture

## Abstract

Sex identification and the selection of monosex male tilapia are crucial for tilapia aquaculture. In this study, we evaluated the application of ultrasonography as an alternative procedure for sex identification and reproductive assessment in Nile tilapia (*Oreochromis niloticus*). Ultrasonography was performed on 23 fish weighing 232–1,281 g to capture longitudinal and transverse images of the ovaries and testes. Female tilapia were identified by the presence of numerous small egg granules and gray or light gray ovarian tissue and male tilapia by the homogeneous echogenicity and uniform gray tubular appearance of the testes. These distinct echogenic patterns allowed for accurate differentiation between the female and male fish. Ultrasonography achieved a 95% accuracy in sex identification, with minimal stress to the fish during the procedure, compared to manual sex sorting, which demonstrated an accuracy of 87%. Furthermore, the method facilitated the assessment of the reproductive status of the fish, including the maturation stages and fecundity potential. The use of ultrasonography offers significant advantages over traditional methods, such as animal welfare enhancements and improved breeding program efficiency. Our findings support the integration of ultrasonography into aquaculture practices and will promote sustainable and humane fish farming while optimizing reproductive management and productivity.

## 1 Introduction

Nile tilapia is a widely cultured and economically important species of fish. It plays a significant role in food security, especially in developing countries, with an estimated annual production value of 7 billion US dollars (1, 2). The increasing demand for tilapia for consumption over the past few decades has led to an expansion in fry and fingerling production and broodstock development (3). Meanwhile, improvements in various aspects of hatcheries, such as breeding programs for fast-growing fish, the selection of only male fingerlings, the management of fish health, and the development of vaccines, are approaches that aim to promote the sustainability of the tilapia aquaculture industry (4). Nevertheless, there remains a need for improved breeding programs that depend on accurate sex determination to inform selective breeding program and population management. Indeed, the optimal male-to-female ratio for effective tilapia reproduction is generally one male to two to four females (5, 6), as a single male can produce sufficient sperm to fertilize the eggs of several females (7). Accurate sex selection is therefore essential for precise production planning, ensuring that fry output aligns with production goals.

The conventional method currently used for sex selection in tilapia relies on an examination of the fish genitalia, which is heavily dependent on the experience of the workers and can lead to errors. In fact, manual sex sorting based on external genitalia has an accuracy rate ranging from 80% to 90%, which results in a high ratio of misidentified fish (8–10). Sex differentiation in tilapia can be undertaken on farms when the fish are 4–6 months old and have an average body length of 20–30 cm (11). In female fish, there are three openings: urinary pores for urine excretion, a genital pore, which is the relatively large opening for egg release from the oviduct, and the anus, which is the opening for excreting feces. In male fish, the sex can be identified by observing two openings: the urogenital pore, from which sperm and urine are excreted, and the anus. Moreover, the male genital papilla has a cone-like shape and is located behind the anus (8, 9, 12).

Ultrasonography is a diagnostic imaging technique in which high-frequency sound waves are used to produce real-time images of internal organs, tissues, and blood flow (13, 14). The technique has been successfully established in various animal species to monitor gonadal development, assess reproductive status, and detect abnormalities of the reproductive organs (15–20). Specifically, ultrasonography can be used to monitor the spawning status of fish and thus allows for the more efficient management of broodstock (16, 21). Additionally, the technique has been applied for sex identification and to assess the maturity stage of various species, including starry sturgeon (*Acipenser stellatus*) (22), shovelnose sturgeon (*Scaphirhynchus platorynchus*) (23), Russian sturgeon (*Acipenser gueldenstaedtii*) (24), Murray cod (*Maccullochella peelii peelii*) (25), Sichuan taimen (*Hucho bleekeri*) (26), and channel catfish (*Ictalurus punctatus*) (27). Ultrasonography has been utilized to assess the reproductive organs of flathead gray mullet (*Mugil cephalus*) (28), Atlantic salmon (*Salmo salar*) (29–31), and westslope cutthroat trout (*Oncorhynchus clarkii lewisi*) (32). Although ultrasonography has been used in various fish species, it has not yet been applied for sex selection in tilapia or to distinguish between female and male tilapia. Ultrasonography has previously only been used to evaluate carcass yield and to monitor production in tilapia (33, 34).

The objective of this study was to evaluate the application of ultrasonography for sex identification and reproductive assessment in Nile tilapia. By comparing the ultrasound images of the ovaries in females and the testes in males, we aimed to establish a reliable, non-invasive method for sex differentiation and reproductive performance assessment. Our findings could impact the tilapia aquaculture industry by promoting more sustainable and humane practices while enhancing productivity and profitability.

## 2 Materials and methods

### 2.1 Animal and ethics statement

Twenty-five healthy Nile tilapia weighing 232–1,281 g were randomly acquired from a hatchery in Phetchaburi Province, Thailand. Upon arrival, two fish were examined for ectoparasites via skin scraping and gill biopsy, bacterial isolation from the head kidney, and quantitative PCR assays for tilapia lake virus, tilapia parvovirus, and infectious spleen and kidney necrosis virus using a described protocol (35). No ectoparasite, bacteria, and viruses were detected, and the remaining 23 fish were then used in the subsequent experiments. During the experiment, 25% of the water was changed daily, and the water quality parameters, including dissolved oxygen, temperature, ammonia, and nitrite, were monitored every 2 days. The fish were fed commercial feed twice daily and fasted for 24 h before undergoing ultrasound examination. All the animal experiments and procedures were conducted in accordance with the guidelines of and approved by the Kasetsart University Institutional Animal Use Committee (protocol number ACKU67-VET-035).

### 2.2 Anesthesia procedure

The fish were anesthetized using clove oil (Aquanes^®^, Betagro PLC, Bangkok, Thailand) at a concentration of 1 mL per 10 L of water. They were immersed in the anesthetic solution for 5–10 min, and the stage of anesthesia was assessed by the decreased rate of gill operculum movement and loss of balance. During anesthesia, the fish were covered with a wet towel. Their respiratory rate was closely monitored, and a continuous flow of water was maintained over the gills. After completing the procedure, the fish were returned to oxygenated water to facilitate their rapid recovery from the anesthesia.

### 2.3 Sex differentiation and anatomical examination

Before performing ultrasonography, the body weight and length (from the mouth to the tip of the caudal fin) of the individual fish were measured and recorded. The sex of each fish was determined by distinguishing the characteristics of the external genitalia around the genital opening (8). In female fish, there are three openings in the abdominal area: the anus, the genital pore (for egg release from the oviduct), which is relatively large, and the urinary pore. In male fish, there are two openings in the abdominal area: the anus and the urogenital opening (for the excretion of feces, urine, and sperm). The male genitalia are elongated and protrude near the anus.

### 2.4 Ultrasound specification

For the reproductive ultrasound examination, we used the Mindray Vetus E7 (Shenzhen Mindray Animal Medical Technology Co., LTD., Shenzhen, China) and MyLab Alpha (Esaote, Genoa, Italy) ultrasound models. The abdominal area of each fish was examined using a high-definition linear probe with a frequency range of 6.6–13 MHz to generate high-resolution images for accurate sex identification and reproductive status assessment. The depth focus point was maintained at 35–40 mm, and the gain of ultrasound was fine-tuned within a range of 65%−80% to enhance the detail of the captured images. This approach ensured consistency and specificity in the examinations and aligned with standard practices in veterinary ultrasonography.

### 2.5 Reproductive ultrasound imaging procedure

The fish were placed on a wet towel in a right lateral recumbency position. The examination time for each fish was limited to no more than 2 min. The ultrasound probe was positioned from the pectoral fin toward the anterior of the anal fin during sex determination. The images acquired from each fish were recorded in both longitudinal and transverse views for analysis and included the shape, size, and echogenicity patterns of the gonads. All the images were digitized as JPG files and stored for comparative analysis to differentiate between the ovaries in the females and the testes in the males.

### 2.6 Necropsy and calculation of the gonadosomatic index

After performing the reproductive ultrasound, the fish were placed in a highly concentrated clove oil solution (10 mL per 10 L of water) for euthanasia. Death was confirmed by observing the absence of gill operculum movement and the total loss of body movement. Necropsy procedures were employed to accurately identify the sex of the fish and confirm the results of the ultrasonography. During the necropsy process, an incision was made in the abdominal cavity to collect the gonads. The gonadal tissue (ovary or testis) was weighed, and the gonadosomatic index (GSI) was then calculated using the formula (36):

Gonadosomatic index = (gonad weight/total tissue weight) × 100.

### 2.7 Histological examination of ovaries and testes

The gonadal tissues, which included the ovaries and testes of 23 fish, were removed and placed in 10% normal buffered formalin for histological analysis. Following fixation, the tissues underwent dehydration through a series of graded alcohol solutions (70%, 80%, 95%, and 100% ethanol) to remove water and prepare them for embedding in paraffin wax. Subsequently, the samples were transferred into xylene before being embedded in paraffin wax. The tissues were then transversely sectioned at a thickness of 5 μm using a microtome, followed by staining with hematoxylin and eosin. The slides were observed using the VS120^®^ Virtual Microscopy Slide Scanning system (Olympus, Tokyo, Japan) and analyzed with the Olympus OlyVIA 3.1 software (Olympus, Tokyo, Japan). The status of the reproductive organs in the female and male fish was determined by assessing the stage of the oocytes in the ovaries and the sperm in the testes against the assessment criteria provided in Table 1 (36, 37).

**Table 1:** Definition of each stage of the reproductive cycle in female and male tilapia.

**Stage**	**Female**	**Male**
Stage 1: Resting	Thin ovaries with oogonia and perinucleolar follicles	Thin testes with spermatogonia
Stage 2: Ripening	Enlarging ovaries with perinucleolar and previtellogenic follicles	Enlarging testes with germ cells and some spermatozoa
Stage 3: Ripe	Vitellogenic oocytes with germinal vesicle migration to the micropyle, indicating final maturation	Maximum volume testes with spermatozoa in the tubular lumen
Stage 4: Spawned/spent	Flaccid, reduced ovaries with hemorrhagic areas	Emptied, flaccid testes with possible residual spermatozoa

## 3 Results

### 3.1 External appearance of the female and male genitalia

**Figure 1:**
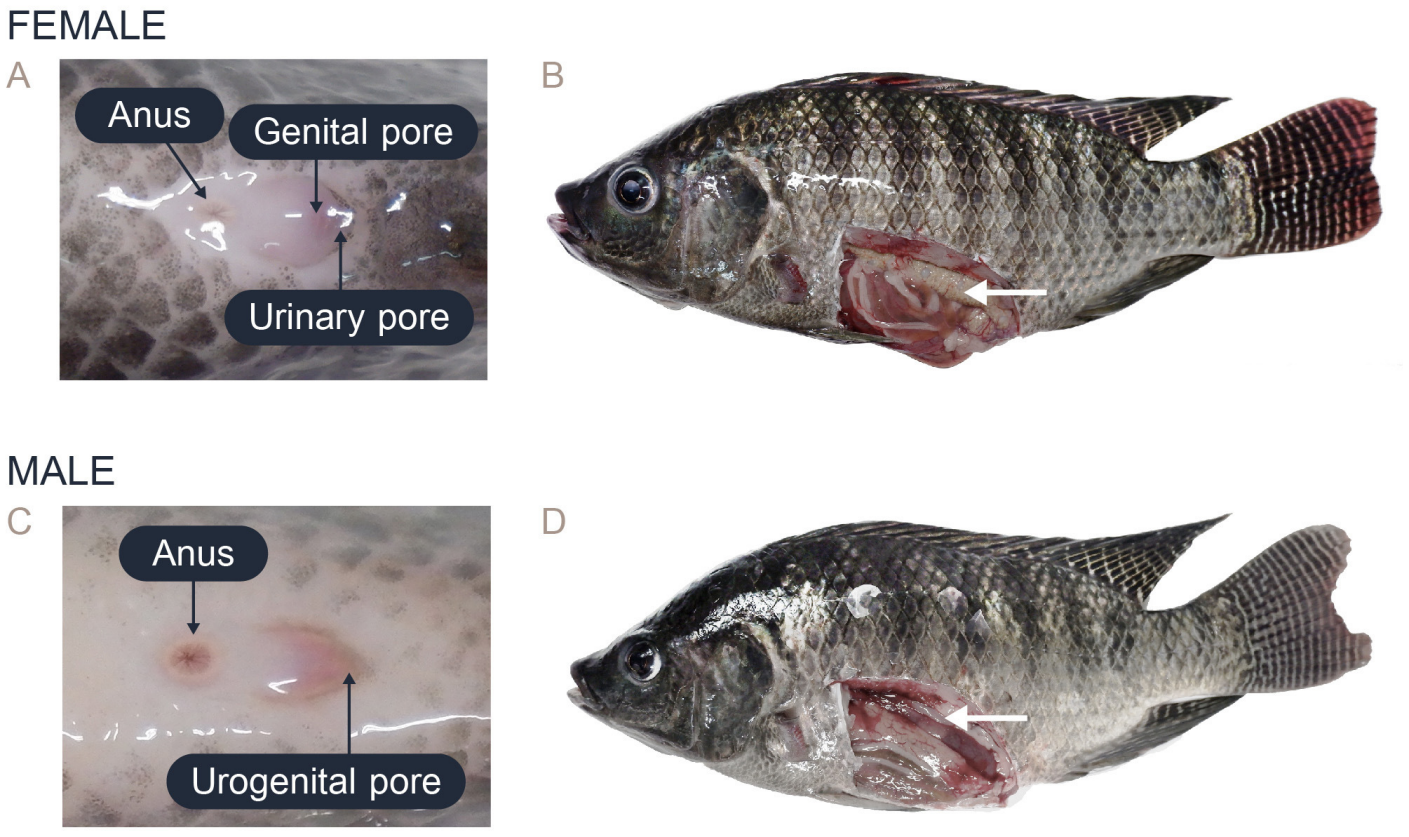
Characteristics of the external genitalia in the posterior area and the gross anatomy of Nile tilapia: **(A)** External genitalia of female Nile tilapia showing the anus, genital pore, and urinary pore. **(B)** Anatomy of the ovary in female Nile tilapia (white arrow). **(C)** External genitalia of male Nile tilapia showing the anus and urogenital opening. **(D)** Anatomy of the testis in male Nile tilapia (white arrow).

In this study, the female Nile tilapia had a mean weight of 511.63 ± 278.90 g and a body length of 29.56 ± 4.4 cm, while the male tilapia had a mean weight of 726.14 ± 371.78 g and a body length of 32.57 ± 4.35 cm (Supplementary Figure 1). Sex identification was determined by analyzing the external morphological characteristics of the fish. The females were distinguished by their oval-shaped external genitalia, which featured three distinct openings: the anus, genital pore, and urinary pore (Figure 1A). During spawning, the females showed abdominal distension and enlargement (Figure 1B). In contrast, the external genitalia of the male fish had a small cone shape with two openings: the anus and urogenital pore (Figure 1C). The testes were located dorsally to the digestive tract within the coelomic cavity, and the appearance and size varied depending on the reproductive stage (Figure 1D).

### 3.2 Placement of probe and ultrasonography

**Figure 2:**
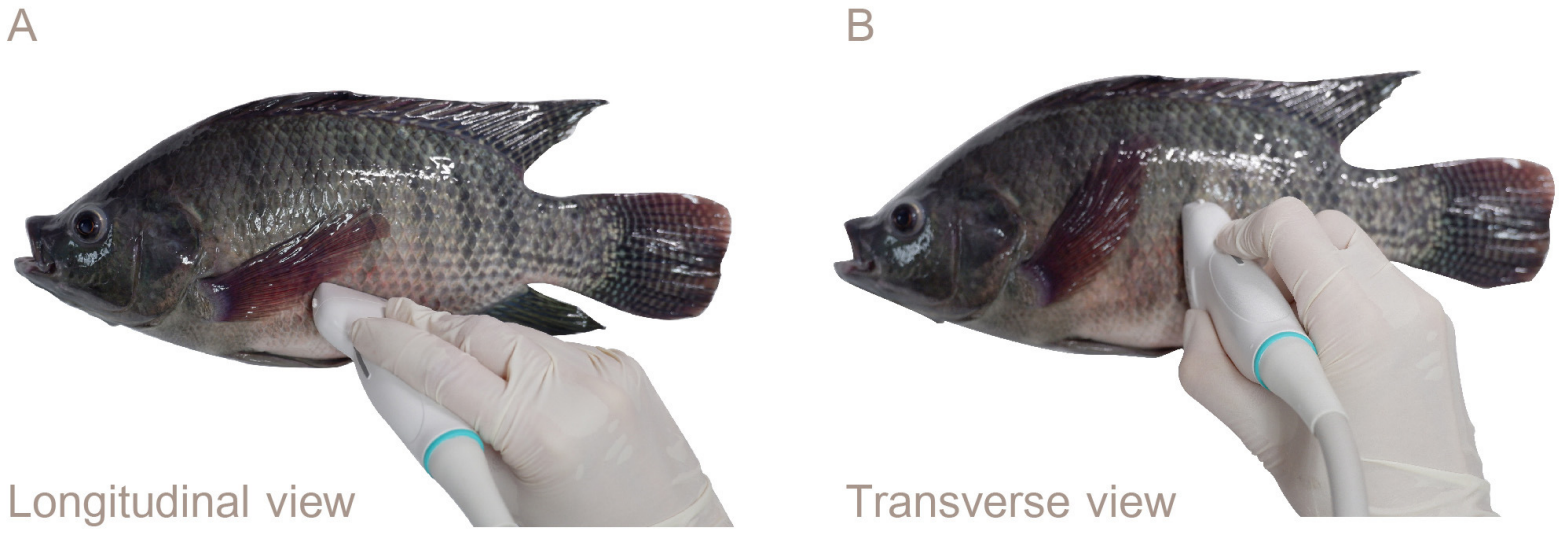
Lateral views showing the transducer placement for ultrasound examination of the reproductive organs of Nile tilapia: **(A)** Left lateral longitudinal orientation. **(B)** Transverse orientation. The genital papilla served as a reference point for reproductive ultrasound in the Nile tilapia.

The fish were placed in right lateral recumbency for the ultrasonography procedure. The ultrasound probe was carefully positioned from the pectoral fin to the anterior of the anal fin using the genital papilla as a key landmark for imaging the reproductive organs. For longitudinal imaging, the transducer was aligned along the left lateral side of the fish parallel to the body length to capture longitudinal views of the reproductive organs (Figure 2A). For transverse imaging, the transducer placement was adjusted on the left lateral side and oriented perpendicular to the body length (Figure 2B).

### 3.3 Ultrasound images, gross morphology, and histology

**Figure 3:**
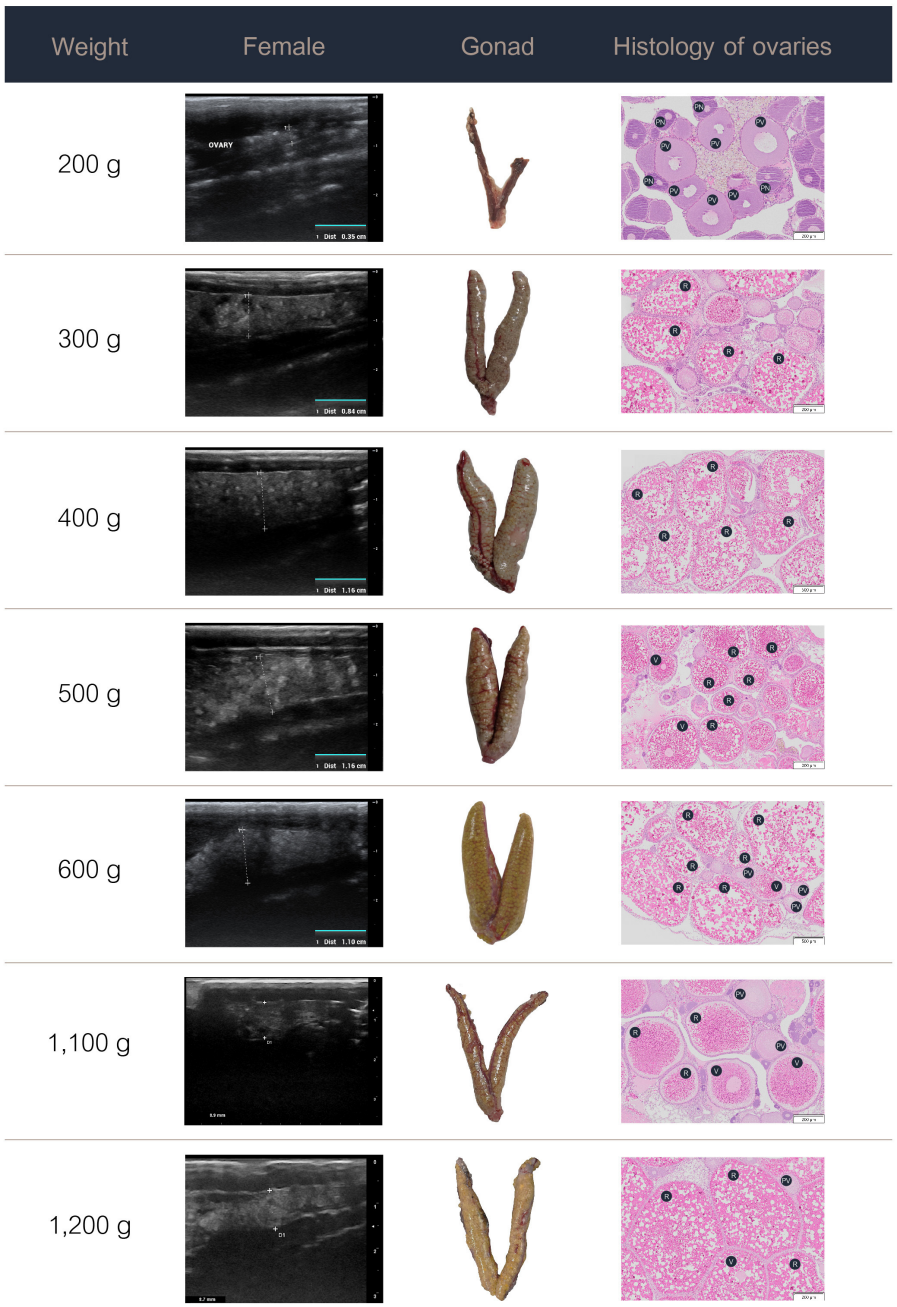
Ultrasound images, gross anatomy, and histology of the ovaries of female Nile tilapia at different weights. PN, perinucleolar oocyte; PV, previtellogenic oocytes; R, ripe-stage oocyte; V, vitellogenic oocyte.

**Figure 4:**
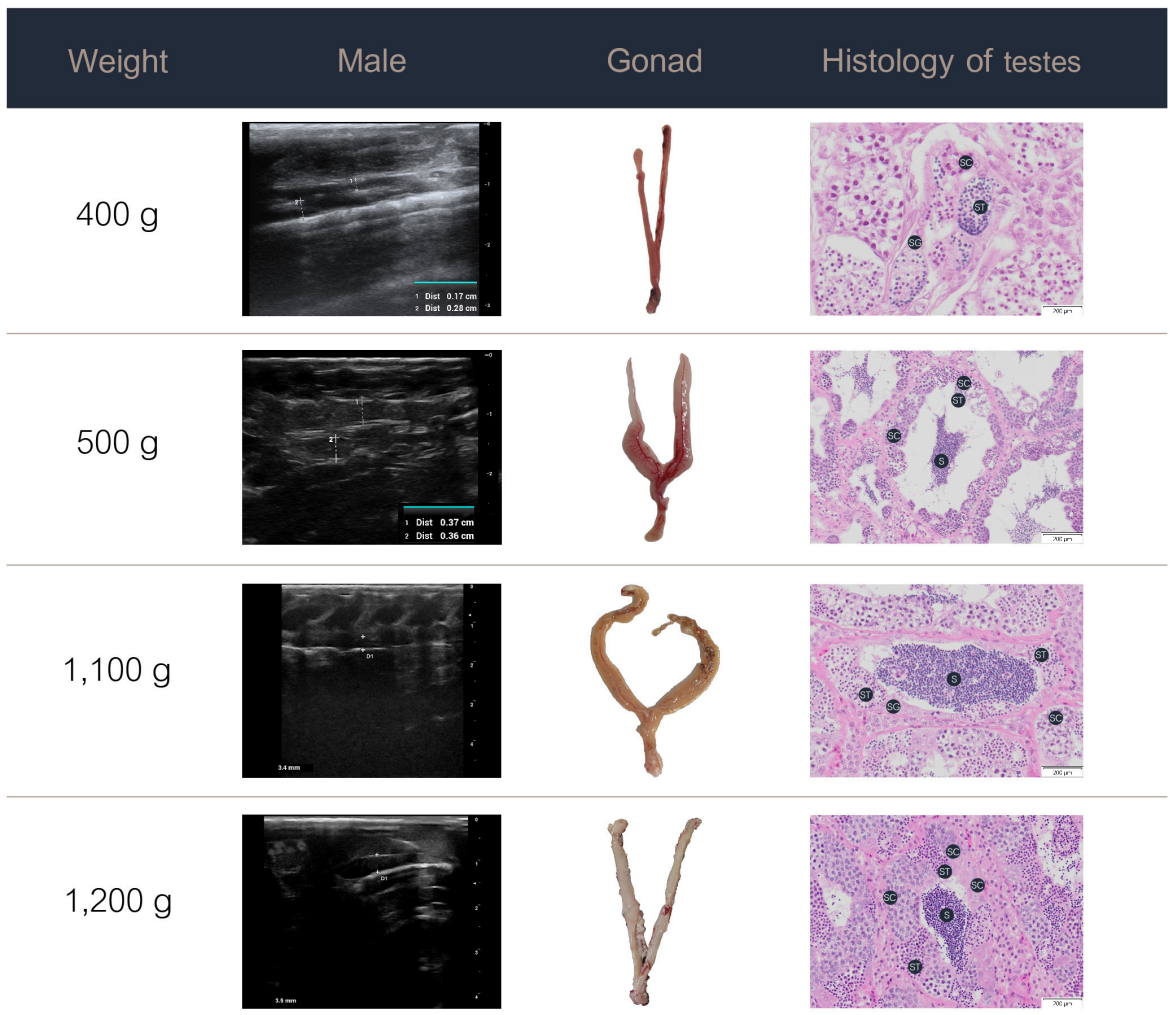
Ultrasound images, gross anatomy, and histology of the testes of male Nile tilapia at different weights. S, spermatozoa; SC, spermatocytes; SG, spermatogonia; ST, spermatids.

The data on the ultrasound images, gross morphology, and histology of the ovaries in the females and testes in the males at various weight stages are presented in Figures 3, 4. Specifically, the progressive development and maturation of the oocytes correlated with increasing body weight in the female Nile tilapia. At a fish weight of 200 g, ultrasonographic imaging showed a thin, small ovary measuring approximately 0.35 cm, with histological sections showing perinucleolar and previtellogenic oocytes characterized by a small size (50–200 μm) and prominent nuclei. For fish weighing 300 g, the ovary appeared larger (0.84 cm) with more distinct oocyte structures, which included increasing numbers of vitellogenic oocytes (450–750 μm) and yolk granules. The female fish weighing 400 g or more had fully grown ovaries measuring over 1 cm in the longitudinal ultrasound views, which indicated the maturation of ripe-stage oocytes ready for egg release. These mature oocytes were larger (850–1,600 μm) than those of the female fish at lower weights and had displaced nuclei and a hydrated yolk.

In the male Nile tilapia weighing 400 g, ultrasound revealed small testes measuring 0.17 cm in the early ripening stage of development. The histological assessment showed spermatogonia, spermatocytes, and limited spermatids in the seminiferous tubules. With increasing body weight of 500 g and above, the testes measured over 0.4 cm on ultrasound and showed numerous germ cell cysts and wider lumens filled with mature sperm. During the spent stage, the seminiferous tubules contained germ cells and a partially emptied lumen with a reduced number of spermatozoa.

### 3.4 Analysis of the ultrasound images

**Figure 5:**
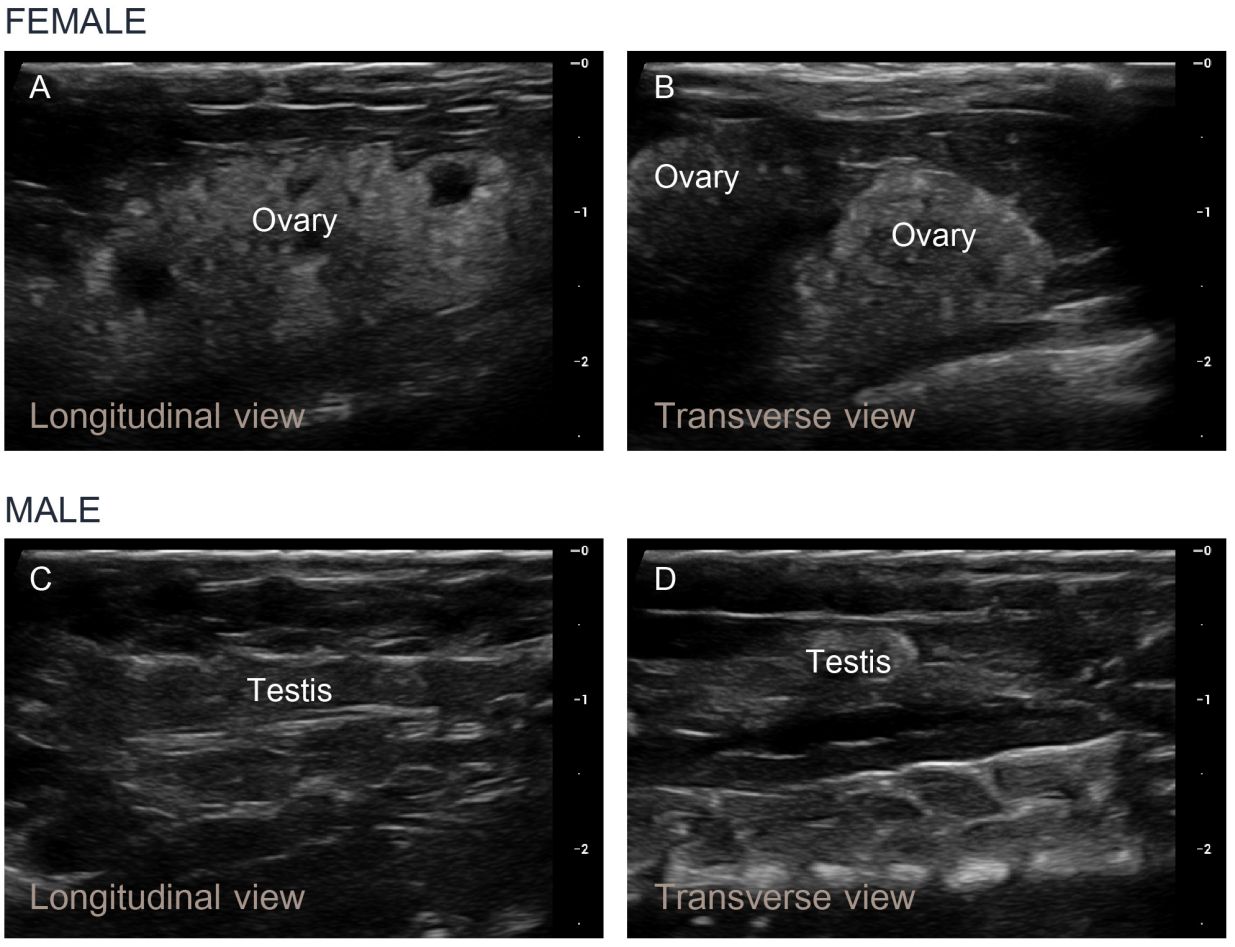
Longitudinal and transverse ultrasound images of the ovaries and testes of Nile tilapia. **(A, B)** In mature female fish approaching the ripe stage, numerous small egg granules were detected within the developing ovaries, and the ovarian tissue appeared light gray. **(C, D)** In mature male fish, homogeneous echogenicity characterized by a uniform gray tubular appearance was observed.

Ultrasonography of the female Nile tilapia in both the longitudinal and transverse orientations successfully detected the ovaries. The ultrasonographic assessments took less than 2 min per fish, with hyperechogenic ovarian structures in ripe-stage mature females being clearly detectable within 30 seconds. The ovarian tissue showed consistent brightness and hyperechogenicity compared to the liver. The numerous oocytes within the ovary appeared as prominent light gray granules on the ultrasound images (Figure 5A). In the transverse view, two oval-shaped ovarian structures were observed with clearly visible and distinct oocytes, which was consistent with the findings in the longitudinal view (Figure 5B). In the male tilapia, both the longitudinal and transverse views showed homogeneous echogenicity characterized by a uniform gray tubular appearance (Figures 5C, D). These findings confirmed the application of ultrasound imaging for sex identification in Nile tilapia based on the echogenic patterns and anatomical structures of their reproductive organs. However, in the male Nile tilapia weighing < 400 g, the small size of the testes presented challenges, which led to less precise ultrasound measurements.

### 3.5 Sex identification and gonadosomatic index

The results of the sex identification method are presented in Table 2. Necropsy, which is considered the definitive technique for sex identification, demonstrated higher consistency with the ultrasonographic determinations than the genital papilla examination. Ultrasonography achieved a 95% accuracy rate, with correct identification in 22 of the 23 fish. In contrast, manual sex sorting based on external genitalia was accurate in 20 fish (87% accuracy rate). All the fish for which the sex was incorrectly identified using both genital papilla examination and ultrasound weighed < 500 g. These inaccurately identified fish comprised 17% of the total sample size (4 of the 23 fish).

**Table 2:** Data for the sex determination for each Nile tilapia via genital papilla examination, ultrasonographic sex determination, necropsy, fork length, weight, gonad weight, and gonadosomatic index.

**Individual number**	**Sex determination from genital papilla**	**Ultrasonographic sex determination**	**Necropsy**	**Fork length (cm)**	**Weight (g)**	**Weight of gonad (g)**	**Gonadosomatic index**
1	Female	Female	Female	39	1,102	7.6	0.69
2	Male	Male	Male	39	1,281	2.6	0.2
3	Female	Female	Female	40	1,236	10	0.81
4	Male	Male	Male	38	1,227	3.4	0.28
5	Female	Female	Female	29	517	0.6	0.12
6	Male	Male	Male	28	516	0.1	0.02
7	Male	Male	Male	33	694	0.1	0.01
8	Female	Female	Female	30	622	2.1	0.34
9	Female	Female	Female	32	621	17.9	2.88
10	Female	Female	Female	26	232	0.3	0.13
11	Female	Female	Female	28	364	0.4	0.11
12	Female	Female	Female	26	349	0.7	0.2
13	Female	Male	Female	24	337	0.3	0.09
14	Female	Female	Female	28	383	3.3	0.86
15	Female	Male	Male	29	403	0.5	0.12
16	Female	Male	Male	30	461	0.6	0.13
17	Female	Female	Female	25	310	1.3	0.42
18	Female	Female	Female	29	438	16.5	3.77
19	Female	Male	Male	31	498	4.4	0.88
20	Female	Female	Female	31	511	10	1.96
21	Female	Female	Female	29	387	7.5	1.94
22	Female	Female	Female	30	433	13.6	3.14
23	Female	Female	Female	27	344	15.1	4.39

The female gonad weights ranged from 0.3 g to 17.9 g, while the male gonad weights ranged from 0.1 g to 4.4 g. An analysis of the gonadosomatic index (GSI) data from the female and male Nile tilapia revealed values ranging from 0.09 to 4.39 for the females, with higher GSI values indicating a more mature reproductive stage. In contrast, the male GSI values were lower (0.01–0.88). The GSI values measured for each Nile tilapia indicated varying stages of reproductive maturity. Furthermore, the Nile tilapia with GSI values below 0.1 consistently showed male characteristics based on ultrasound examination.

## 4 Discussion

Ultrasonography is a valuable diagnostic tool in veterinary medicine due to its non-invasive technique for assessing reproductive health and sex identification. In this study, we demonstrated the applicability of ultrasonography as a reliable tool for sex identification and reproductive assessment in Nile tilapia. The distinct echogenic patterns observed in the ovaries and testes provided fundamental information to distinguish between the female and male fish, respectively. Ultrasonography presents advantages over traditional techniques, such as manual sex sorting based on external genitalia, which has shown 87% accuracy compared to the 95% accuracy achieved with ultrasonography and subsequently confirmed by the necropsy results in this study. In practice in farm operations, the effectiveness of manual sex sorting by examining the external genitalia is highly dependent on the skill and experience of the examiner (9). Thus, if the examiner lacks sufficient experience, inaccuracies in sex identification can occur, which can complicate broodstock management and fry production (8, 10). Indeed, combining manual sorting methods with ultrasound could potentially increase the accuracy of sex identification without compromising animal welfare under standard farming conditions. Previous studies have indicated that the application of ultrasound for sex determination in fish is highly accurate, with rates ranging from 82.6% to 100% (22, 25, 28, 38). Although most ultrasound studies have been conducted on fish weighing over 1 kg (22–24, 26–31), the application of this technique for sex identification and internal organ assessment in smaller fish, such as Nile tilapia, remains a remarkable challenge. Multiple factors, including fish size, age, maturity stage, and equipment quality, can affect the accuracy of ultrasound interpretations (16, 28, 38). The limitations observed in this study, particularly the difficulty in identifying small testes in male Nile tilapia weighing less than 400 g, highlight the need for improvements in ultrasonographic techniques to enhance accuracy in smaller fish. Adjustments to the frequency and depth settings of the ultrasound machine, as well as improving the sensitivity of the equipment, are crucial. For example, higher frequency sound waves can provide better visualization of internal and reproductive organs in smaller fish (39). Further research should focus on these technological improvements to expand the applicability of ultrasonography across different fish sizes and species.

The approach to ultrasound probe positioning is important and varies depending on the anatomy of the fish. For species such as Sichuan taimen, catfish, Japanese ornamental carp, salmon, and trout, the probe is typically placed on the lateral side of the fish, similar to the technique used for the Nile tilapia in this study (23, 25–27, 29, 30). In contrast, species with a fusiform body shape, such as flathead gray mullet and sturgeon, require dorsal recumbent positioning with the probe placed directly on the ventral surface (28, 40). Determining the appropriate probe positioning for different fish species should therefore improve the accuracy of ultrasound analysis. The ultrasound imaging in this study was more consistent in the mature adult fish, as the echogenic patterns in the reproductive organs were clear. Similar to the findings for flathead gray mullet, our study highlighted the limitations of using ultrasonography to detect small testes, which are influenced by fish size (28). Comparable challenges have been noted in studies on salmon, where thin testes are present in early stage testicular development and are not easily visible on echogram, which can complicate sex determination using ultrasonography (41).

The GSI is an important indicator of reproductive status and particularly beneficial in identifying spawning periods. The GSI is measured by the ratio of gonad weight to total body weight and provides insights into the reproductive cycle and seasonal spawning (36). Typically, female fish show higher GSI values due to their heavier gonads. The increase in ovary weight before spawning is a notable characteristic in gravid females, which makes the GSI an essential parameter for assessing reproductive maturity (42, 43). The combination of ultrasound with GSI measurements can thus enhance the accuracy of reproductive assessments. The integration of ultrasonography in aquaculture practices holds considerable potential for optimizing breeding programs and improving overall fish production (16). Furthermore, ultrasound imaging can be applied as a non-invasive procedure, as it can be performed while the fish remain in water and under mild anesthesia (16, 27), which will reduce stress in the fish and support animal welfare principles (29, 30, 44). Additionally, the ability to conduct repeated assessments allows for continuous monitoring of the reproductive cycle, which can facilitate timely interventions and improve the management of breeding stocks.

In terms of applying this technique, future research should focus on using ultrasonographic methods in different ways, for example, to examine the ovary size of fish to determine its correlation with the GSI and to study ultrasonographic characteristics at different developmental stages (31, 38, 45). Developing an automated ultrasonography-based system for sex differentiation in fish could facilitate rapid and accurate sex identification, enabling the effective identification of male and female fish. This improvement could benefit commercial breeding operations by improving efficiency, reducing labor costs, and minimizing human error. These approaches have the potential to improve the management of not only Nile tilapia but also other important fish species. Nevertheless, there remains a significant knowledge gap concerning the use of ultrasound to detect abnormalities in reproductive organs and to diagnose pathological conditions in the liver, gallbladder, heart, and other internal organs of fish (41, 46). Overall, ultrasonography is an existing tool that can be used for non-invasive sex determination and reproductive assessment in Nile tilapia. This technique supports sustainable and effective breeding programs and can contribute to the overall productivity and management of tilapia hatcheries. Moreover, applying this technique to other species may benefit the sustainability of the aquaculture industry, which is facing increasing challenges.

## 5 Conclusion

In our study, we demonstrated the applicability of ultrasonography as a non-invasive tool for sex identification and reproductive assessment in Nile tilapia. The echogenic patterns observed in the ultrasound images of the ovaries and testes enabled effective differentiation between the female and male fish, respectively. These findings are consistent with previous research and confirm the application of ultrasound for monitoring gonadal development and reproductive health in tilapia aquaculture. Importantly, integrating ultrasound technology in fish farming practices has the potential to enhance selective breeding programs, improve production efficiency, and promote sustainable aquaculture. Future research should focus on refining ultrasound techniques and exploring their application in other commercially important fish species to further validate their utility in the industry.

## Data Availability

The datasets presented in this article are not readily available because the data that support the findings of this study are available from the corresponding author upon reasonable request. Requests to access the datasets should be directed to win.s@ku.th.
