# Supplementary material for: Ultrasonography for non-invasive sex identification and reproductive assessment in Nile tilapia (Oreochromis niloticus)
Source: Front Vet Sci. 2024 Oct 9;11:1467158. doi: 10.3389/fvets.2024.1467158 (PMC11497460; doi:10.3389/fvets.2024.1467158)
Supplement: Supplementary file 1 [file Image_1.pdf]

Supplementary Materials

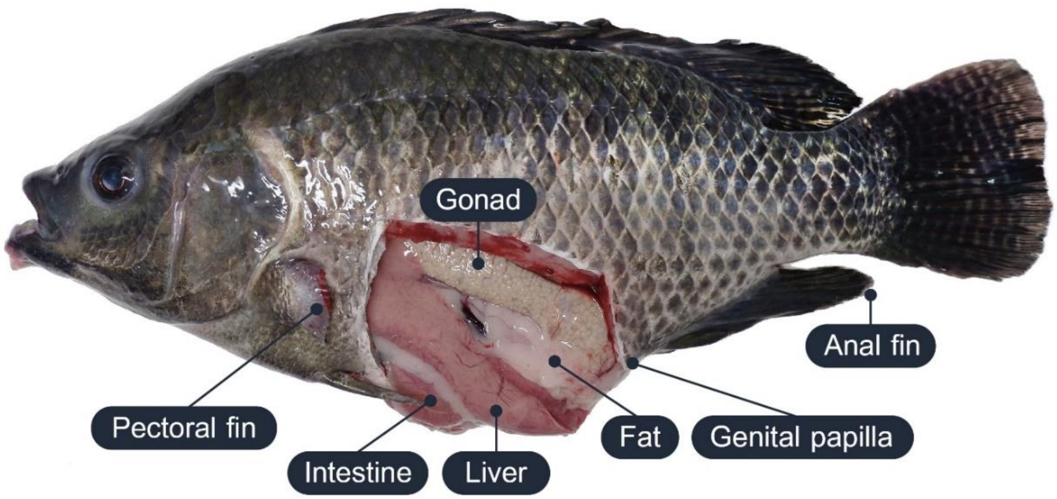

Supplementary Figure 1. The anatomy and internal organs of a Nile tilapia.
